# Syntrophic Partners Enhance Growth and Respiratory Dehalogenation of Hexachlorobenzene by *Dehalococcoides mccartyi* Strain CBDB1

**DOI:** 10.3389/fmicb.2018.01927

**Published:** 2018-08-22

**Authors:** Anh T. T. Chau, Matthew Lee, Lorenz Adrian, Michael J. Manefield

**Affiliations:** ^1^College of Agriculture and Applied Biology, Cantho University, Can Tho, Vietnam; ^2^School of Civil and Environmental Engineering, University of New South Wales, Sydney, NSW, Australia; ^3^Department Isotope Biogeochemistry, Helmholtz Centre for Environmental Research – UFZ, Leipzig, Germany; ^4^School of Chemical Engineering, University of New South Wales, Sydney, NSW, Australia

**Keywords:** syntrophy, hexachlorobenzene, organohalide respiration, *Dehalococcoides mccartyi* strain CBDB1, carbon monoxide

## Abstract

This study investigated syntrophic interactions between chlorinated benzene respiring *Dehalococcoides mccartyi* strain CBDB1 and fermenting partners (*Desulfovibrio vulgaris, Syntrophobacter fumaroxidans*, and *Geobacter lovleyi*) during hexachlorobenzene respiration. Dechlorination rates in syntrophic co-cultures were enhanced 2-3 fold compared to H_2_ fed CBDB1 pure cultures (0.23 ± 0.04 μmol Cl^−^ day^−1^). Syntrophic partners were also able to supply cobalamins to CBDB1, albeit with 3–10 fold lower resultant dechlorination activity compared to cultures receiving exogenous cyanocobalamin. Strain CBDB1 pure cultures accumulated ~1 μmol of carbon monoxide per 87.5 μmol Cl^−^ released during hexachlorobenzene respiration resulting in decreases in dechlorination activity. The syntrophic partners investigated were shown to consume carbon monoxide generated by CBDB1, thus relieving carbon monoxide autotoxicity. Accumulation of lesser chlorinated chlorobenzene congeners (1,3- and 1,4-dichlorobenzene and 1,3,5-trichlorobenzene) also inhibited dechlorination activity and their removal from the headspace through adsorption to granular activated carbon was shown to restore activity. Proteomic analysis revealed co-culturing strain CBDB1 with *Geobacter lovleyi* upregulated CBDB1 genes associated with reductive dehalogenases, hydrogenases, formate dehydrogenase, and ribosomal proteins. These data provide insight into CBDB1 ecology and inform strategies for application of CBDB1 in ex situ hexachlorobenzene destruction technologies.

## Introduction

Chlorinated benzenes including hexachlorobenzene (HCB) are toxic and persistent compounds that have been studied extensively in the context of microbiological degradation (Field and Sierra-Alvarez, 2008). Whilst lesser chlorinated benzenes are susceptible to aerobic biodegradation in conventional activated sludge treatment processes (van Agteren et al., 1998) microbes can only degrade hexachlorobenzene, pentachlorobenzene, 1,2,3,4-tetrachlorobenzene, and 1,3,5-trichlorobenzene through anaerobic or reductive reactions (Adrian and Görisch, 2002). Such reductive reactions have been linked to bacterial growth (Adrian and Görisch, 2002) and therefore have potential as a biotechnology for commercial chlorinated benzene disposal. The world's largest stockpile of HCB (8,000 tons) is maintained in Sydney, Australia.

To date, only two genera, *Dehalococoides* and *Dehalobacter* are known to contain species that use HCB as an electron acceptor in a respiratory process. *Dehalococcoides mccartyi* strains are obligate hydrogenotrophs and are restricted to organohalides as electron acceptors. *D. mccartyi* strain CBDB1 grows in mineral medium with H_2_ as the electron donor, acetate as an organic carbon source, and chlorobenzenes as electron acceptors, in conjunction with cyanocobalamin as an essential cofactor (Adrian et al., 2000).

Syntrophic interactions between fermentative bacteria and organohalide respiring bacteria (ORB) are of interest because they underpin dechlorination reactions in organochlorine contaminated environments where H_2_ is generated via fermentation of organic matter. The term syntrophy has been used to describe microbial cross-feeding as a cooperation where both partners are involved in metabolic activity and cannot be replaced by adding a co-substrate or any type of nutrient (Schink, 1997). The benefits that syntrophic growth in co-culture can offer over a pure culture include the generation of a metabolic resource by one microbe serving as a resource for another and enhancing the growth/metabolism of one microbe by preventing accumulation of inhibitory compounds consumed by another (McInerney et al., 2008; Morris et al., 2013; Worm et al., 2014). Specifically, in the context of this study, the principal mode of syntrophy involves H_2_ produced by fermentation of reduced organic substrates serving as an electron donor for hydrogenotrophic ORB that in turn maintain low H_2_ partial pressures enabling fermentation to proceed (Becker et al., 2005).

Despite the importance of syntrophy in ecology and engineering settings, data on syntrophic interactions involving ORB remain limited. Breakthrough studies in this area focused on syntrophic growth of a dechlorinating organism (DCB-1), a benzoate degrader (BZ-1), and a lithotrophic methanogen (*Methanospirillum* strain PM-1) in dechlorination of 3-chlorobenzoate (Dolfing and Tiedje, 1986) and methanogenic incubation with *Syntrophus* species degrading 3-chlorobenzoate and 2-chlorophenol (Becker et al., 2005). Research more closely related to the current study focused on syntrophic growth of chlorinated ethene respiring *D. mccartyi* strain 195 with fermenters *Desulfovibrio desulfuricans* and/or *Acetobacterium woodii* (He et al., 2007), interspecies corrinoid transfer between *Geobacter lovleyi* and *D. mccartyi* strains BAV1 and FL2 (Yan et al., 2012, 2013) and interspecies cobamide transfer from the methanogen *Methanobacterium congolense* to *D. mccartyi* (Men et al., 2012).

Carbon monoxide (CO) was recently discovered to inhibit chlorinated ethene dechlorination of *D. mccartyi* strain 195 but the presence of a syntrophic partner was shown to mitigate this toxicity and enhance the growth as well as dechlorination activity (Zhuang et al., 2014; Mao et al., 2015). Additionally, organochlorine dechlorination products can inhibit dechlorination activity (Wei et al., 2016). To date, the inhibitory impacts of lesser chlorinated ethenes alone have been investigated for such inhibition (Yu and Semprini, 2004; Yu et al., 2005; Popat and Deshusses, 2011; Wang et al., 2014; Jiang et al., 2015).

In this study syntrophic interactions of HCB respiring *D. mccartyi* strain CBDB1 and fermentative partners during reductive dechlorination of HCB were investigated, with a view to utilizing syntrophic co-cultures or enrichment cultures in *ex situ* remediation technologies for HCB destruction where full-scale deployment of gaseous hydrogen is considered a safety hazard. Three syntrophic partners were examined, namely *Desulfovibrio vulgaris, Geobacter lovleyi*, and *Syntrophobacter fumaroxidans* provided with lactate, acetate and propionate respectively as carbon and energy sources. *D. mccartyi* cannot derive energy from these organic substrates and none of the syntrophic partners can utilize HCB creating syntrophic dependencies.

This is the first study to reveal syntrophic interactions in HCB respiration between strain CBDB1 and fermenters. CO auto-toxicity to strain CBDB1 is described along with the impact of CO consumption by syntrophic partners. Daughter product inhibition is also described along with a demonstration of the impact of removing lesser chlorinated chlorobenzenes from culture headspace. The data generated broadens our understanding of syntrophic metabolism in organohalide respiration by *D. mccartyi* and provides information relevant to bioreactor applications for HCB destruction.

## Materials and methods

### Bacterial cultures and growth conditions

*D. mccartyi* strain CBDB1 was grown in a basal medium with 5 mM acetate as described previously (Adrian et al., 1998). Briefly, mineral salts medium was amended with a vitamin solution, SL-9 minerals and Trace B mineral solution (Adrian et al., 1998). Final concentrations of 100 mM NaHCO_3_ solution was used to buffer the media to pH 6.8–7.0. Aliquots (80 mL) were dispensed into 160 mL serum flasks and flushed with N_2_ gas, sealed with Teflon septa and sterilized at 121°C for 20 min. After cooling Ti (III) citrate solution was amended to 1 mM and 0.2 bar of N_2_:CO_2_ (4:1, vol/vol) and 0.3 bar of H_2_ were added to the headspace. Hexachlorobenzene (20 mg, Sigma-Aldrich) was added as electron acceptor and H_2_ as electron donor with nominal concentration of 7.5 mM as described previously (Jayachandran et al., 2003).

*Desulfovibro vulgaris* (DSM 2119) and *Syntrophobacter fumaroxidans* (DSM 10017) were obtained from the German Collection of Microorganisms (DSMZ) and grown according to DSMZ recommendations. Briefly, *D. vulgaris* was grown in the medium described above with 10 mM lactate as carbon and energy source and 10 mM sulfate as electron acceptor. *S. fumaroxidans* was grown in MPOB medium with 20 mM sodium fumarate as described previously (Harmsen et al., 1998). *Geobacter lovleyi* was isolated by dilution to extinction from a chlorinated ethene contaminated aquifer in Sydney, Australia using acetate and Fe (III) citrate as electron donor and acceptor respectively. Purity was confirmed through microscopy and molecular fingerprinting and the species was identified by sequencing the near full length 16S rRNA gene (98% similarity to *G. lovleyi* strain SZ).

Co-cultures containing *D. mccartyi* strain CBDB1 with *D. vulgaris* (DSV/CBDB1), with *S. fumaroxidans* (SFO/CBDB1), or with *G. lovleyi* (GBL/CBDB1) were grown in the media described above for strain CBDB1 with the substitutions of 10 mM lactate, 30 mM propionate or 30 mM acetate, respectively as electron donor with no exogenous H_2_ added. Cyanocobalamin (1 μM VB_12_) was added as indicated. All cultures were inoculated to an total initial density of ~5 × 10^6^ cells mL^−1^ and incubated in the dark at 30°C without agitation.

### Application of granular activated carbon (GAC) to mitigate HCB daughter product toxicity

GAC (0.5 g, G60 powder, 100 mesh, Sigma-Aldrich) was mounted in glass Pasteur pipettes sealed at one end and placed into CBDB1 co-cultures such that the sealed end was submerged and the open end exposed to the headspace enabling exposure of GAC to the headspace but avoiding direct contact between GAC and the culture medium (Figure S3). Thus volatile organic substances (lesser chlorinated benzenes) could be adsorbed from the headspace. To quantify trichlorobenzene (TCB) and dichlorobenzene (DCB) removal from the headspace, GAC was extracted five times using 3 mL of dichloromethane (DCM). DCM was reduced to a final volume of 1 mL via evaporation prior to analysis by gas chromatography with flame ionization detection (GC-FID). Extraction recovery was determined to be 90-95% (data not shown).

### Quantification of chlorinated benzenes

Chlorinated benzenes were quantified by headspace analysis gas chromatography using a Shimadzu GC-2010 Plus gas chromatograph with flame ionization detection (GC-FID) fitted with a J&W DB-5 30 m × 0.32 mm (inner diameter) × 0.25 μm column and a Shimadzu headspace auto-sampler (Koutsogiannouli et al., 2009). Culture (1 mL) was transferred into a 10 mL headspace sample vial. Prior to injection, the samples were heated to 80°C and shaken for 2 min. The injector and detector temperature were set at 250°C. The oven temperature was held at 100°C for 1 min, followed by a 25°C min^−1^ increase, and then held at 250°C for 0.5 min. Aqueous chlorinated benzene standards were prepared with the same gas to liquid space ratio as the cultures flasks accounting for phase partitioning according to Henry's Law. The amount of chloride released was calculated by multiplying the concentration of daughter products (mmole l^−1^) by the difference in Cl^−^ number between the parent and the daughter compounds.

### Carbon monoxide quantification

Carbon monoxide (CO) was quantified with a Shimadzu GC-2010 Plus GC, equipped with a pulsed discharge detector (GC-PDD) and fitted with a J&W Molecular sieve (30 m × 0.32 mm (inner diameter) × 0.25 μm column (Wurm et al., 2003). Helium was used as the carrier gas with a split ratio of 1:10. The oven was held at 30°C for 1.5 min, ramped at 20°C min^−1^ to 50°C, then held for 7.5 min. The samples were analyzed with the PDD at 140°C. Aliquots (100 μL) of sample headspace were manually injected with a gas tight syringe. CO standards (0.5–12 μmol) were prepared in 80 mL of CBDB1 media in 160 mL flasks.

### Quantitative polymerase chain reaction (qPCR)

Liquid samples (2 ml) were harvested by centrifugation (8000 × g, 20 min at 4°C). Genomic DNA was extracted from cell pellets as described previously (Urakawa et al., 2010). qPCR was used to determine *Dehalococcoides* spp. and total Bacterial 16S rRNA gene copies in the cultures using *Dehalococcoides* specific primers Dco728F (5′-AAGGCGGTTTTCTAGGTTGTCAC- 3′) and Dco994R (5′-CTTCATGCATGTCAAAT-3′) (Smits et al., 2004), and universal bacterial primers Eub1048F (5′-GTGSTGCAYGGYTGTCGTCA-3′) and Eub1194R (5′-ACGTCRTCCMCACCTTCCTC-3′) (Horz et al., 2005). Reaction mixtures (10 μL final volume) contained 5 μL SsoFast Eva Green Supermix (BioRad), 100 nM of each forward and reverse primer, 2 μL template, 0.1 mg bovine serum albumin (Thermo Fisher Scientific) and 2.7 μL nuclease free water (Thermo Fisher Scientific). For total bacteria quantification, cycling conditions on a CF96 Real Time System (BioRad) were as follows: 3 min at 98°C, 39 cycles of 0.2 min at 95°C and 0.5 min at 62°C, followed by melt curve analysis from 60 to 99°C. For *Dehalococcoides*, cycling conditions were as follows: 3 min at 98°C, 44 cycles of 0.3 min at 94°C and 0.45 min at 58°C, followed by melt curve analysis from 55 to 95°C. Quantification of total Bacteria and *Dehalococcoides* 16S rRNA gene copies was performed by analyzing serial dilution of known quantities of plasmids containing partial *Dehalococcoides* spp. genes.

### Microbiological cobalamin assay

To quantify extracellular cobamides in pure cultures and co-cultures, a microbiological assay using the cobamide-auxotroph *Lactobacillus delbrueckii* (ATCC7830) was performed as described previously (Yan et al., 2012). Cells from 1.5 mL of culture were removed by centrifugation at 14,000 xg for 1 min, and the supernatants were passed through 0.22 μm membrane filters. Cell-free supernatant (150 μl) was diluted from 2- to 20-fold with water and distributed into 96 well plates filled with 150 μl of double-strength vitamin B_12_ assay medium (Difco). To prepare vitamin B_12_-free inocula, cell pellets were washed with sterile, deionized water three times. The cells were suspended in double-strength vitamin B_12_ assay medium diluted with an equal amount of sterile, deionized water and then incubated at 37°C for 3 h to deplete carryover VB_12_ from the inoculum broth. The 96 well plate was sealed with an adhesive optical cover and incubated at 37°C in the dark. After 24 h of incubation, the optical density in each well was recorded at 630 nm using a 96 well plate reader (BioTek, Winooski, VT). A standard curve generated by adding known concentrations of vitamin B_12_ was included on each plate. The assay had a linear range from 5 to 50 ng/L of vitamin B_12_ with a detection limit of 2 ng/L. Cobamides measured using this microbiological approach were reported as vitamin B_12_ equivalents.

### Estimation of the limits of interspecies distance for H_2_ transfer

The maximum interspecies distances between strain CBDB1 and syntrophic partners that would enable the observed dechlorination rates for substrates fermentation were estimated using Fick's diffusion law according to the procedure described by Mao et al. (2015):

$$\begin{array}{l} {\text{J}}_{\text{H}2}={\text{A}}_{\text{s}yn}\times {\text{D}}_{\text{H}2}\times \frac{{\text{C}}_{H2-\text{syn}-{\text{C}}_{\text{H}2}-\text{CBDB}1}}{{\text{d}}_{\text{syn}-\text{CBDB}1}} \end{array}$$

J_H2_ is the flux of H_2_ between syntrophic cells and CBDB1, A_syn_ is the surface area of a syntrophic cell, D_H2_ is the H_2_ diffusion constant in water, at 35°C (6.31 × 10^5^ cm^2^ s^−1^, Haynes, 2013). C_H2−syn_ is the H_2_ concentration immediately outside syntroph cells (representing the maximum H_2_ concentration enabling exergonic fermentation). C_H2−CBDB1_ is the H_2_ concentration immediately outside CBDB1 cells (representing the theoretical minimum H_2_ concentration that strain CBDB1 can use for energy generation from HCB dechlorination). d_syn−CBDB1_ is the distance between syntroph and CBDB1 cells that enables syntrophic oxidation.

### Proteomics

*D. mccartyi* CBDB1 was grown with 5 mM acetate, 7.5 mM H_2_ and excess HCB as described above. *Geobacter lovleyi* was grown with 10 mM acetate and 20 mM Fe (III) citrate. *D. mccartyi* CBDB1 and *G. lovleyi* co-culture (CBDB1/GL) was grown in the same medium as strain CBDB1 pure culture, with 30 mM acetate and no exogenous H_2_. All cultures were grown in triplicate in 100 mL media. Cells were collected from pooled replicates (x3) during exponential growth when cell density reached ~10^8^ cell/mL (~90 days of incubation). Cells were harvested using 0.2 μm filters (Millipore, Germany) as described previously (Schiffmann et al., 2016). Cells were washed from filters with 50 mM ammonium hydrogen carbonate. Three analytical replicates were analyzed for each sample.

Samples were prepared for in-solution digestion as described previously (Schiffmann et al., 2014). CBDB1 cells were lysed by three cycles of freeze (liquid nitrogen) and thaw (1 min at 40 °C). The protein lysates obtained were reduced with 50 mM dithiothreitol (Invitrogen, USA) for 1 h at 30°C and alkylated using 130 mM iodoacetamide (Sigma-Aldrich, Australia) for 1 h at 30°C. Proteolysis was performed overnight using trypsin at 37°C. The tryptic digestion was stopped with formic acid to a final concentration of 1% followed by desalting using ZipTip-μC18 tips and re-suspended in 1% formic acid, 2% acetonitrile (ACN), prior to LC–MS/MS analysis.

Digested peptides were separated by nano-LC using an Ultimate nano RSLC UPLC and auto-sampler system (Dionex, Amsterdam, Netherlands) as described previously (Jugder et al., 2016). Samples (2.5 mL) were concentrated and desalted onto a micro C18 pre-column (300 mm × 5 mm, Dionex) with 2% ACN in water with 0.1% triflouroacetic acid (TFA) at 15 ml/min. After a 4 min wash the pre-column was switched (Valco 10 port UPLC valve, Valco, Houston, Tx) into line with a fritless nano column (75 m × ~15 cm) containing C18AQ media (1.9 μ, 120 Å Dr Maisch, Ammerbuch-Entringen Germany). Peptides were eluted using a linear gradient of 2%−36% ACN in water containing 0.1% (v/v) formic acid at 200 nl/min over 30 min. High voltage (2000 V) was applied to low volume Titanium union (Valco) with the column oven heated to 45°C (Sonation, Biberach, Germany) and the tip positioned ~0.5 cm from the heated capillary (T = 300°C) of a QExactive Plus (Thermo Electron, Bremen, Germany) mass spectrometer. Positive ions were generated by electrospray and the QExactive operated in data dependent acquisition mode (Yang et al., 2010). A survey scan m/z 350-1750 was acquired (resolution = 70,000 at m/z 200, with an accumulation target value of 1,000,000 ions) and lockmass enabled (m/z 445.12003). Up to the 10 most abundant ions (> 80,000 counts, underfill ratio 10%) with charge states > +2 and < +7 were sequentially isolated (width m/z 2.5) and fragmented by HCD (NCE = 30) with an AGC target of 100,000 ions (resolution = 17,500 at m/z 200). M/z ratios selected for MS/MS were dynamically excluded for 30 s. Peak lists generated were submitted to the database search program MASCOT (version 2.5.1, Matrix Science). All MS/MS spectra were searched against a custom database consisting of all proteins in *D. mccartyi* strain CBDB1 and *G. lovleyi* genomes from Uniprot. Search parameters were: Precursor tolerance 4 ppm and product ion tolerances ±0.05 Da; Met(O) carboxyamidomethyl-Cys specified as variable modification, enzyme specificity was trypsin, 1 missed cleavage was possible and the non-redundant protein database from Uniprot (Jan 2015) searched. The results were exported to both XML and ROV file format prior to loading into Scaffold for further analysis.

Label free quantification was carried out in Scaffold Q1 software (version Scaffold 4.7.2, Proteome Software) according to the precursor intensity-based method, where differences in protein abundance (defined here as proteins differentially expressed relative to total cell protein) between pure culture and co-culture samples were found based on the average precursor intensity acquired. Protein identifications were accepted if they could be established at greater than 95.0% probability and contained at least 2 identified peptides. Protein probabilities were assigned by the Protein Prophet algorithm (Nesvizhskii et al., 2003). Proteins that contained similar peptides and could not be differentiated based on MS/MS analysis alone were grouped to satisfy the principles of parsimony. Proteins sharing significant peptide evidence were grouped into clusters.

### Statistical tests

The data was assumed to be normally distributed. One-way analysis of variance (ANOVA) tests and *T*-test were calculated to determine the significance of results using an alpha level of 0.05. Multiple comparisons were calculated with Dunnet's test, where applicable. All statistical tests were performed using GraphPad Prism 6 software.

## Results

### Syntrophic growth of *D. mccartyi* strain CBDB1 and syntrophic partners

Three bacterial strains were selected as hydrogen producing syntrophic partners for *D. mccartyi* strain CBDB1 based on the diversity of substrates used. The partner organisms included Deltaproteobacteria *Desulfovibrio vulgaris* (DSV) and *Geobacter lovleyi* (GBL) and Firmicute *Syntrophomonas fumaroxidans* (SFO). These organisms were supplied with lactate, acetate and propionate respectively as electron donors. Syntrophic growth of *D. mccartyi* strain CBDB1 and H_2_ producing partners was established enabling reductive dechlorination of hexachlorobenzene (HCB) to 1,3,5-trichlorobenzene (1,3,5-TCB), 1,3-dichlorobenzene (1,3-DCB), and 1,4-dichlorobenzene (1,4-DCB) without exogenous H_2_ provision.

Dechlorination rates were comparable with the different partners with the highest observed in the *D. vulgaris* and CBDB1 (DSV/CBDB1) co-culture (0.59 ± 0.05 μmol Cl^−^ day^−1^) with the rates in the other co-cultures at 0.39 ± 0.15 and 0.45 ± 0.09 μmol Cl^−^ day^−1^ in the *S. fumaroxidans* and CBDB1 (SFO/CBDB1) and *G. lovleyi* and CBDB1 (GBL/CBDB1) co-cultures, respectively (Table 1). The dechlorination rates in all co-cultures were significantly higher (2-3 fold, *t*-test, *P* < 0.05) compared to pure H_2_/acetate fed CBDB1 culture (0.23 ± 0.04 μmol Cl^−^ day^−1^).

**Table 1:** Maximum dechlorination rate and cell yield of *D. mccartyi* in syntrophic growth with syntrophic partners.

**Co-culture**	**Dechlorination rate** ** (μmol Cl^−^day^−1^)**	**CBDB1 cell yield** ** (cells μmol^−1^ Cl^−^day^−1^)**	***P*-value** ** (*T*-test)**
DSV/CBDB1	0.59 ± 0.05^***^	3.7 (±0.3) × 10^8^	0.0005
SFO/CBDB1	0.39 ± 0.12^*^	2.7 (±0.1) × 10^8^	0.048
GBL/CBDB1	0.45 ± 0.09^**^	3.8 (±0.8) × 10^8^	0.003
CBDB1	0.23 ± 0.04	2.3 (±0.3) × 10^8^	

P-values for rates. Significantly higher than CBDB1 culture alone at

* P < 0.05,

** P < 0.01,

*** *P < 0.001. Values are given as means ± the standard deviation from triplicate cultures*.

**Figure 1:**
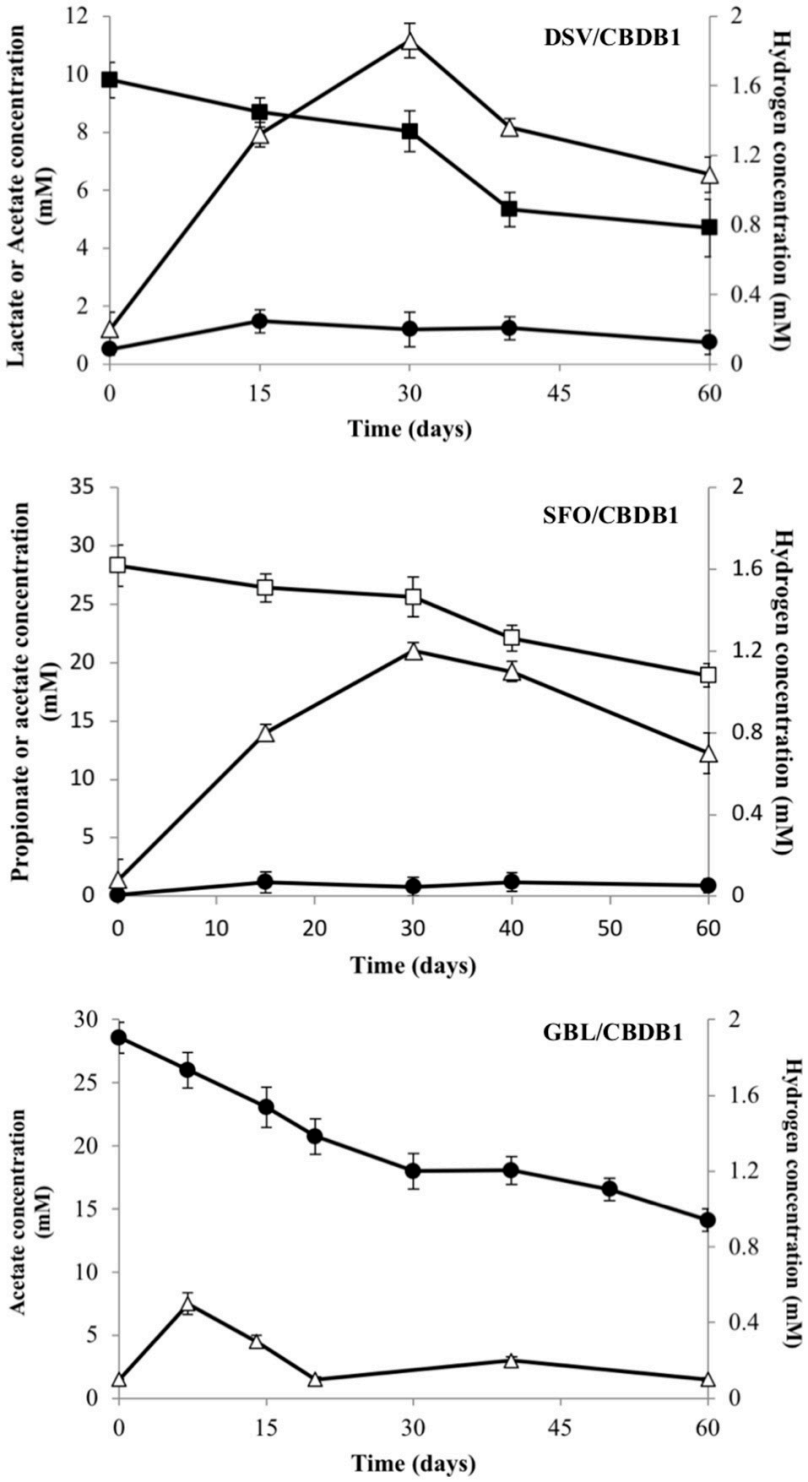
Electron donor production and consumption in HCB reducing syntrophic co- cultmes DSV/CBDB1, SFO/CBDB1 and GBL/CBDB1. H2 (triangles), Acetate (circles), Lactate (closed squares), Propionate (open squares). Error bars represents standard deviation of the mean (*n* = 3).

Acetate and H_2_ accumulated to a maximum of 1.8 (±0.06) mM and 2.0 (±0.09) mM respectively in the DSV/CBDB1 co-culture, while in the SFO/CBDB1 co-culture accumulation was slightly lower with 1.1 (±0.02) mM of acetate and 1.2 (±0.03) mM of H_2_ observed (Figure 1). Thereafter, acetate and H_2_ concentrations decreased, presumably as a function of consumption by CBDB1. In the acetate fed GBL/CBDB1 co-culture, acetate consumption corresponded with H_2_ concentration peaking at 0.5 (±0.01) mM followed by consumption and stabilization of H_2_ concentration and ongoing acetate consumption (Figure 1). After two months incubation, 5.1 (±0.41), 9.4 (±0.98), and 13.7 (±0.94) mM lactate, propionate and acetate had been consumed respectively.

Growth of *D. mccartyi* strain CBDB1 in co-culture was measured by quantitation of 16S rRNA genes using universal bacterial and *Dehalococcoides* specific primers. Cell numbers increased over 90 days. Initial 16S rRNA gene copy concentrations were 2.0 (±0.15) × 10^6^ and 1.2 (±0.4) × 10^6^ copies/mL for total bacteria and *Dehalococcoides* respectively. Deltaproteobacterial co-cultures DSV/CBDB1 and GBL/CBDB1 had the largest increase in both total bacterial gene copies and *Dehalococcoides* gene copies (110–125 fold) up to 2.2 (±1.2) × 10^8^ and 1.5 (±1.8) × 10^8^ copies /mL, respectively. The SFO/CBDB1 co-culture showed the lowest *Dehalococcoides* cell growth up to 8.4 (±0.3) × 10^7^ copies/mL (70-fold increase) congruent with dechlorination activity data.

### Cell to cell distance and thermodynamic considerations in syntrophic co-cultures

The maximum interspecies distance for molecular hydrogen transfer between cells of strain CBDB1 and syntrophic partners can be calculated using Fick's diffusion law as previously described for other *D. mccartyi* strains (Mao et al., 2015). This distance was calculated for the syntrophic co-cultures of strain CBDB1 and partners with lactate, propionate and acetate as substrates. In the randomly dispersed cells of DSV/CBDB1, GBL/CBDB1 and SFO/CBDB1 co-cultures, the average distance enabling interspecies H_2_ transfer was calculated to be 178, 10 and 19 μm, respectively (Table 2). The values for cell-cell distances in DSV/CBDB1, GBL/CBDB1 and SFO/CBDB1 co-cultures calculated using 16S qPCR data for bacteria as proxy were 16.8, 19.5, and 24.4 μm, respectively. The measured value for the DSV/CBDB1 co-culture was well below the theoretical maximum whilst the GBL/CBDB1 and SFO/CBDB1 co-cultures had measured values marginally above the theoretical maximum. This potentially explains the superior activity of the lactate fed DSV/CBDB1 co-culture across all quantified metrics (dechlorination activity, growth and acetate and H_2_ production).

**Table 2:** Estimation of the cell-cell distance in syntrophic co-culture at day 60 of incubation.

	***D. vulgaris* with strain CBDB1 on lactate**	***G. lovleyi* with strain CBDB1 on acetate**	***S. fumaroxidans* with strain CBDB1 on propionate**
Total number of cells/ml ^(a)^	2.11 × 10^8^	1.34 × 10^8^	6.89 × 10^7^
Mean cell-cell distance (μm) ^(b)^	16.8	19.5	24.4
Maximum interspecies distance for H_2_ transfer (μm) ^(c)^	178	10.1	18.6

*(a) Total cell number for strain CBDB1 and syntrophic partners in co-cultures using 16S copies as proxy; (b) Calculated using 16S copies as a proxy for cell number; (c) Calculated using Fick's diffusion law for H_2_ transfer from syntrophic partners to strain CBDB1. Detailed calculations for these values can be found in the Supplementary Materials Table S1*.

### Effect of syntrophic partners on CO concentrations in CBDB1 co-cultures

**Figure 2:**
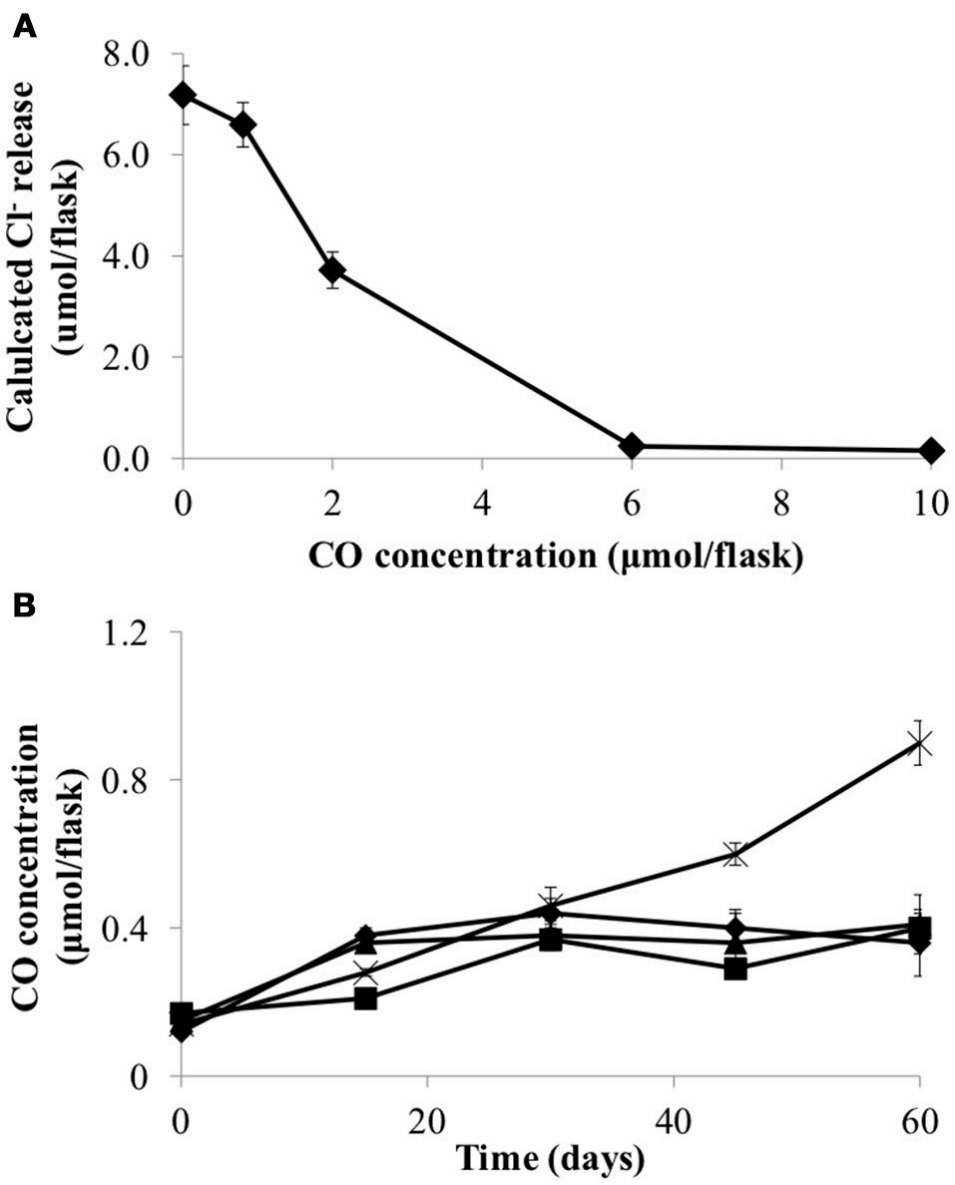
**(A)** CO inhibits HCB dechlorination activity of *D. mccartyi* strain CBDB1 shown as Cl- ion release at the end of incubation (60 days) and **(B)** CO accumulation during HCB dechlorination by strain CBDB1 in pure culture (crosses) and in co-cultures with *D. vulgaris* (DSV/CBDB1, diamonds), *S. fumaroxidans* (SFO/CBDB1, squares) and *G. lovleyi* (GBL/CBDB1, triangles). After 60 days strain CBDB1 produced enough CO to confer autotoxicity. In co-cultures, CO concentration remained below that showing toxicity. Data points are averages of hiplicate cultures. Error bars represent the standard deviation (*n* = 3).

Carbon monoxide (CO) has previously been shown to be inhibitory to *D. mccartyi* strain 195 (Zhuang et al., 2014). Consequently, CO toxicity, production and consumption was examined in strain CBDB1. Figure 2 shows that CO concentrations above 1 μmol flask^−1^ reduce the dechlorination activity of strain CBDB1 and 6 μmol flask^−1^ is completely inhibitory. The half maximal inhibitory concentration (IC50) was 2.24 ± 0.09 μmol flask^−1^. Figure 2 also shows that after 60 days incubation, CO accumulation in strain CBDB1 pure culture reached the threshold whereby CO toxicity manifests (0.9 ± 0.1 μmol flask^−1^). After 60 days, 17% of the initial HCB amendment (70 μmol) was degraded to release 11.2 ± 0.2 μmol Cl^−^. Hence, complete dechlorination of 70 μmol HCB could accumulate ~5.6 μmol CO in pure culture. In syntrophic co-culture, regardless of the fermentative partner, the CO concentration remained below those shown to confer toxicity at 0.4 μmol flask^−1^ in all co-cultures after 60 days (Figure 2).

### Growth of CBDB1 with syntrophic partners excluding exogenous cobalamin supply

**Figure 3:**
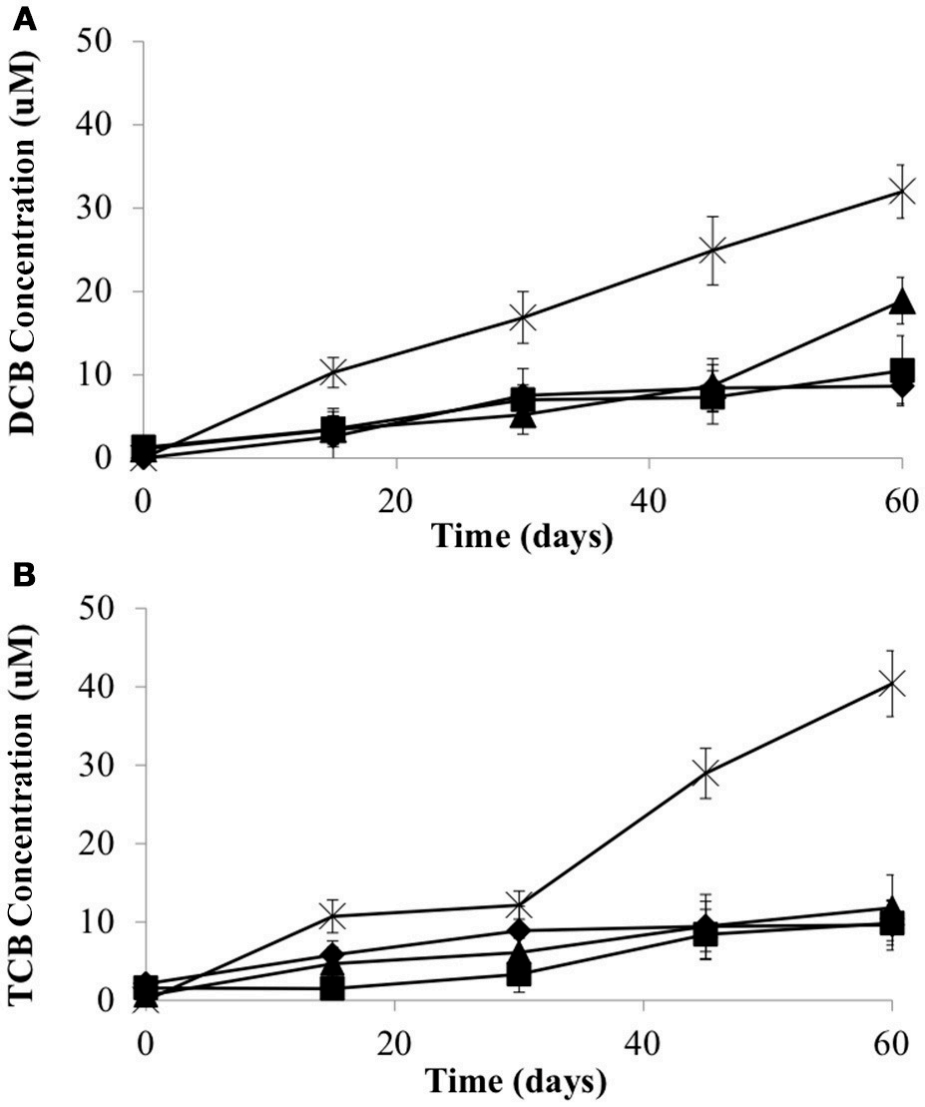
Production of **(A)** 1,3-Dichlorobenzene and 1,4-Dichlorobenzene (sum total) and **(B)** 1,3,5-Trichlorobenzene from HCB by *Dehalococcoides mccartyi* strain CBDB1 in pure culture with exogenous provision of acetate, H_2_ and Vitamin B_12_ (crosses), and in co-culture with *D. vulgaris* (diamonds), S. *fumaroxidans* (squares) or *G. lovleyi* (triangles) without exogenous provision of acetate, H_2_ and Vitamin B_12_. Data points are averages of triplicate cultures. Error bars represent standard deviation.

To examine whether the syntrophic partners could supply cobalamin to CBDB1, microcosms were established without exogenous cyanocobalamin. Data was collected after four transfers (10% v/v inoculation) of the parent culture representing a dilution of cobalamin from 0.5 μg/L to 0.05 ng/L. The total concentration of HCB dechlorination products were on average 28.3 ± 1.8 μM in all three co-cultures, compared to 72.4 ± 3.5 μM in pure CBDB1 culture (supplied with cyanocobalamin) at day 60 (Figure 3). Dechlorination activity in co-cultures was ten-fold less than in co-cultures amended with cyanocobalamin and three-fold less than a pure CBDB1 culture. Dechlorination was not observed in a control where CBDB1 pure cultures were not supplied exogenous cobalamin (data not shown).

Measurement of cobamides in co-culture supernatants was achieved using a biological assay involving the growth of the cobamide auxotroph *Lactobacillus delbrueckii*. Supernatants were analyzed at the end of the experiment (i.e., day 60). In GBL/CBDB1 cultures 6.0 ± 1.0 ng/L extracellular cobamide was detected. In the DSV/CBDB1 and SFO/CBDB1 co-cultures extracellular cobalamins were below the limits of detection. This suggests that none of the partners could produce sufficient cobalamin to support maximum HCB dechlorination activity by *D. mccartyi* strain CBDB1 (ie., activity observed with cyanocobalamin amendment).

### HCB respiration products inhibit *D. mccartyi* strain CBDB1 growth and activity

**Figure 4:**
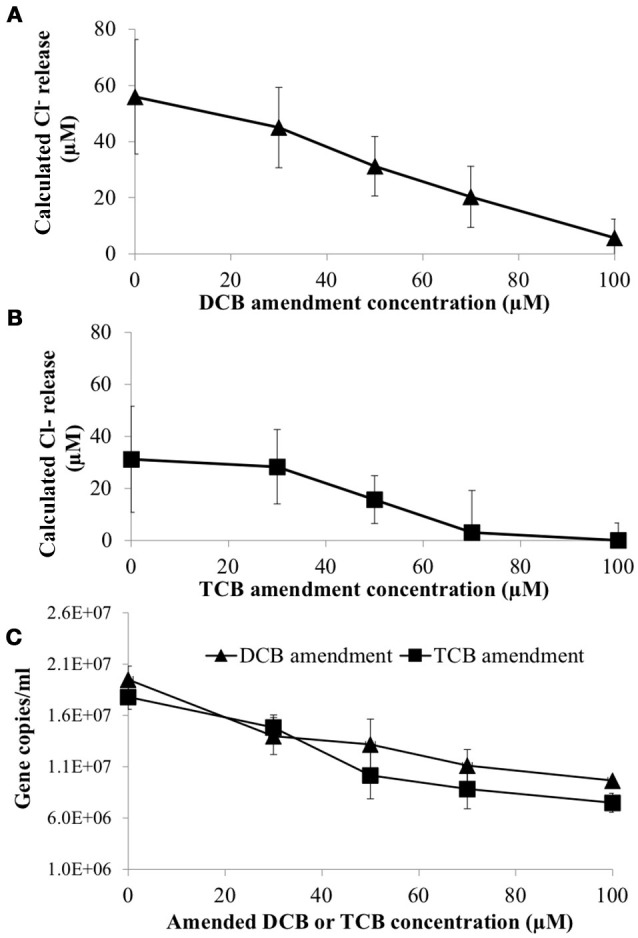
Impact of daughter products from reductive dechlorination of HCB (Dichlorobenzene or Trichlorobenzene) on HCB reduction activity of *Dehalococcoides mccartyi* CBDB1 after 30 days incubation. **(A)** Chloride ion release calculated from concentrations of DCB and TCB produced from HCB in the presence of increasing amended DCB concentrations. **(B)** Chloride ion release calculated from concentrations of DCB and TCB produced from HCB in the presence of increasing TCB concentrations. **(C)** Impact of DCB and TCB on CBDB1 gene copy concentration after 30 days incubation. Error bars represent SD (*n* = 3).

To determine if 1,3- and 1,4-dichlorobenzene (DCB) and 1,3,5-trichlorobenzene (TCB) had an inhibitory impact on HCB respiration by *D. mccartyi* strain CBDB1, pure cultures were established with increasing concentrations of DCB or TCB. Figure 4 shows that DCB and TCB have an inhibitory effect on HCB dechlorination activity and cell growth of CBDB1, with lower IC50 and LD50 values for TCB compared to DCB (45 μM and 70 μM, respectively).

DCB and TCB are volatile compounds so Granular Activated Carbon (GAC) was mounted in culture flask headspace (Figure S3) in an attempt to mitigate the inhibitory impacts of daughter product formation from HCB reduction. First order rate constants for DCB and TCB adsorption on GAC were −0.14 (±95% CI) day^−1^ and −0.16 (±90% CI) day^−1^, respectively (Figure S1). In comparison, first order rate constants of DCB and TCB production in strain CBDB1 culture were 0.1 (±99% CI) day^−1^ and 0.07 (±97% CI) day^−1^, respectively. Hence, trapping DCB and TCB on GAC could mitigate the inhibitory effect of DCB and TCB accumulation CBDB1 in co-cultures.

**Figure 5:**
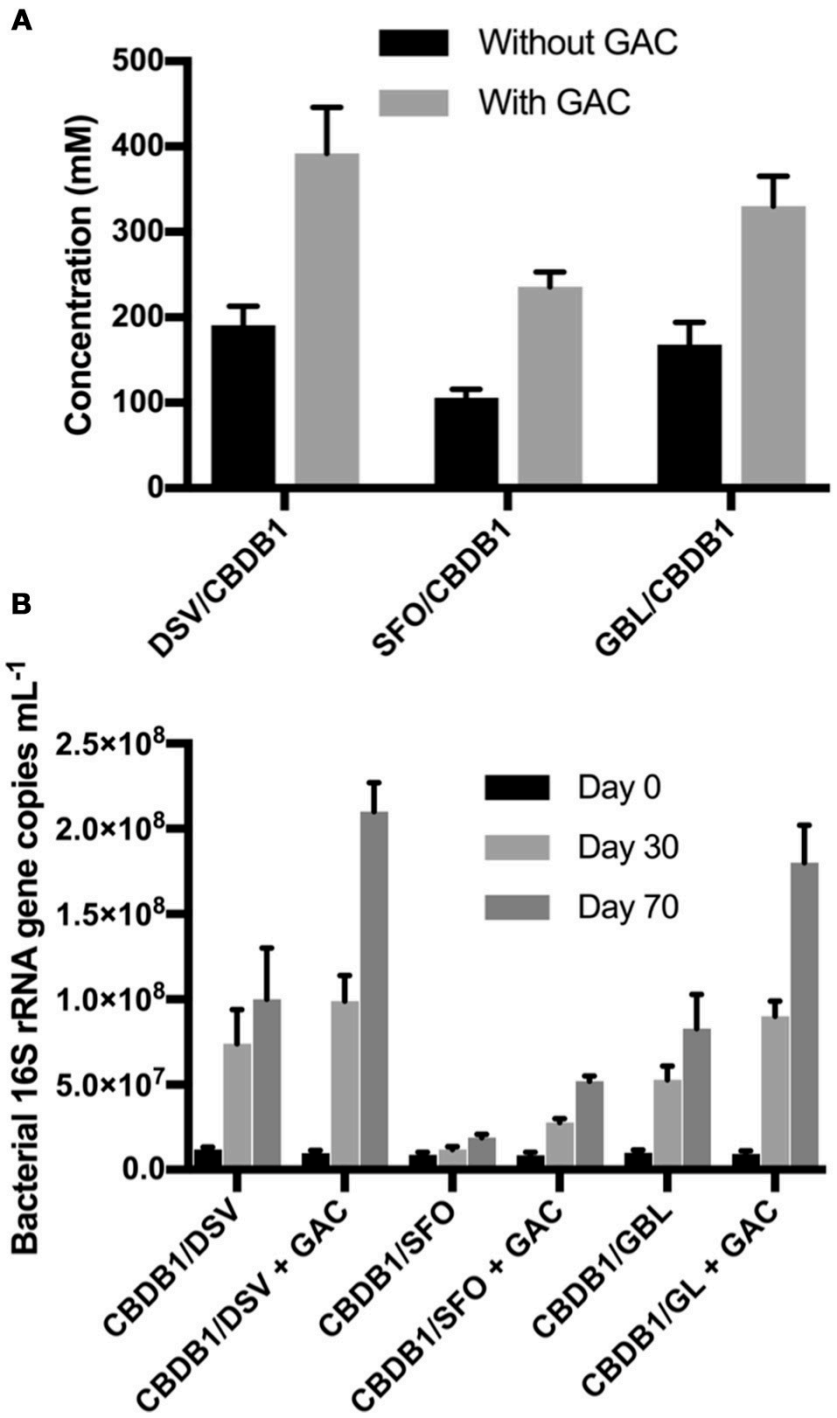
**(A)** Total concentrations of HCB dechlorination products (1,3- and 1,4-DCB and 1,3,5-TCB) in different co-cultures of *D. mccartyi* strain CBDB1 with and without GAC after 70 days of incubation. Data points are averages of triplicate cultures. Error bars represent the standard deviation (*n* = 3). **(B)** Quantification of 16S rRNA gene copies in syntrophic co-cultures of *D. mccartyi* strain CBDB1 and partners with and without GAC after 70 days of incubation. All cultures were provided with cyanocobalamin, Ti (III) citrate, and without exogenous H_2_. Data points are averages of triplicate cultures. Error bars represent the standard deviation.

To test the mitigation strategy, DSV/CBDB1, SFO/CBDB1 and GBL/CBDB1 co-cultures were grown with and without 0.5 g GAC mounted in the culture headspace. At day 70, HCB daughter products were analyzed both in the culture medium and adsorbed to GAC. The latter was analyzed by dichloromethane (DCM) extractions of the GAC. DCB and TCB production were significantly higher (2-fold, *t*-test, *P* <0 .05) in co-cultures amended with GAC for all three syntrophic partners (Figure 5). For example, treatments with GAC in DSV/CBDB1 co-cultures had a dechlorination rate of 1.1 ± 0.2 μmol Cl^−^ day^−1^, compared to a rate of 0.6 ± 0.2 μmol Cl^−^ day^−1^ in the GAC-free treatments. Total bacterial growth in treatments with and without GAC was determined via 16S rRNA gene quantitative PCR using universal bacterial primers at day 0, 30 and 70 of incubation. After 70 days the gene copies in the presence of GAC were 2.1-2.7 fold higher (*t*-test, P < 0.05) than in the absence of GAC (Figure 5).

### Proteomic analysis in GL/CBDB1 co-culture

Whole cell proteomic analyses of CBDB1 grown alone and in co-culture with syntrophic partner *G. lovleyi* was undertaken to identify changes in gene expression in response to co-culture. In pure culture *G. lovleyi* was grown with Fe (III) citrate as electron acceptor. Equal amounts of total protein (~30 μg) from late exponential phase samples (~3 months incubation) were digested. Each sample was prepared via the filter aided sample preparation (FASP) method and subjected to LFQ by nano LC-MS/MS. A total of 363 *D. mccartyi* proteins and 404 *G. lovleyi* proteins were identified in pure and co-cultures (Figure S2). Of the *D. mccartyi* proteins, 119 were identified in co-culture with 62 of these exclusively observed in co-culture. Of the *G. lovleyi* proteins, 102 were observed in co-culture with 15 of these exclusive to the co-culture.

Table 3 reports *D. mccartyi* and *G. lovleyi* proteins for which expression was upregulated greater than five-fold (see Table S2 for lesser fold upregulation and Table S3 for the whole data set). Three reductive dehalogenases were identified from *D. mccartyi* including cbdbA84 (accession number CAI82345), cbdbA80 (accession number CAI82340) and cbdbA1453 (accession number CAI83480), which encode 1,2,3- and 1,2,4-TCB reductive dehalogenases. Among them, cbdbA84 and cbdbA80 were more abundant in the co-culture than in the pure CBDB1 culture suggestive of dependence of regulation on the presence of another organism (in this case a syntrophic partner).

**Table 3:** Identified proteins with greater than five-fold increase in abundance in co-culture.

**NCBI locus**	**Predicted functions**	***D. mccartyi***	***G. lovleyi***
cbdbA84	Putative reductive dehalogenase	33	
cbdbA80	Putative reductive dehalogenase	14	
cbdbA195	Formate dehydrogenase, major subunit	8.2	
cbdbA960	Translation elongation factor Tu	5.6	
Glov_0477	Conserved hypothetical protein	–	8.6
Glov_1625	Malate dehydrogenase	–	6.6
Glov_0475	Alkyl hydroperoxide reductase	–	5.5

Translation elongation factor Tu (locus cbdbA960), was among the high coverage proteins that were more abundant in the co-culture than in CBDB1 culture alone. Formate dehydrogenase (locus cbdbA195) was also shown to be more abundant during syntrophic growth than in the CBDB1 isolate. Several other conserved protein complexes involved in respiratory electron transfer were found in the CBDB1 isolate, including Rdh complexes, four hydrogenase complexes (Hup, Ech, Hyc, and Hym), NADH dehydrogenase complexes and two oxidoreductase complexes. Three hydrogenases, VhuA, HymB, and HymC, encoded by cbdbA597, cbdbA684, and cbdbA685, respectively, were found to be marginally more abundant in syntrophic growth than in the isolate (Table S2). The co-culture also possessed 16 ribosomal proteins that were detected with high expression compared to the CBDB1 isolate, congruent with the more robust growth of CBDB1 in co-culture. None of the cobalamin biosynthesis proteins were detected in either the co-culture or the CBDB1 isolate. Conversely, two types of cobalamin transporter, ABC-type cobalamin/Fe^3+^-siderophores transport system ATPase component (locus cbdbA633) and periplasmic component (locus cbdbA636) were detected, with no difference in expression between the co-culture and CBDB1 isolate, both of which were amended with VB_12_.

Among the 87 *G. lovleyi* proteins detected in both *G. lovleyi* culture alone and in co-culture (Figure S2), thiamine pyrophosphate protein (locus Glov_1628), succinate dehydrogenase or fumarate reductase (locus Glov_2213), as well as elongation factor and ribosomal proteins were among the proteins with the highest coverage. Proteins related to acetate oxidation, including malate dehydrogenase (locus Glov_1625), isocitrate dehydrogenase (locus Glov_1624) and citrate synthase (locus Glov_1379), were expressed more abundantly during syntrophic growth than in isolate cultures (Table S2). Two molybdopterinoxidoreductase proteins (loci Glov_3132 and Glov_0661) were detected less abundantly in the co-culture than in the *G. lovleyi* isolate, with fold change ratios of 0.7 and 0.1 respectively.

There were two proteins detected exclusively in the co-culture: peptidylprolylisomerase (locus Glov_2546) and histidinol dehydrogenase (encoded Glov_0822). Interestingly, there were two types of flagellin detected only during *G. lovleyi* growth with Fe (III) citrate, which were the flagellin domain protein (locus Glov_3371) and a flagellin basal associated-protein FilL (locus Glov_3294). In addition, three chemotaxis sensory transducers (encoded Glov_ 2776, Glov_ 1733 and Glov_1239) were found with less abundance during syntrophic growth than during pure culture growth on iron. Cobalamin-dependent proteins were identified in both *G. lovleyi* isolate and co-culture including ribonucleotide reductase, methionine synthase and methylmalonyl-CoA mutase. Among these proteins, only methylmalonyl-CoA mutase (encoded Glov_3260) was more abundant in co-culture than in the isolate, with a fold change of 2.0 (Table S2). Nicotinate-nucleotide/dimethylbenzimidazolephosphoribosyltransferase (CobT-Glov_3082) was the only cobalamin biosynthesis protein detected, which was from *G. lovleyi* in isolation.

## Discussion

In this present study, lactate, propionate and acetate were supplied as organic carbon and energy sources for *D. mccartyi* strain CBDB1 in co-culture with *Desulfovibrio, Syntrophobacter* or *Geobacter*. Oxidation of these substrates by the syntrophic partners of CBDB1 was expected to provide H_2_ for the reductive dechlorination of HCB. Table 4 provides the ΔG^0'^ for the H_2_ producing and H_2_ consuming reactions revealing that H_2_ production in the absence of the H_2_ consuming reductive dechlorination reaction is marginally favorable or unfavorable thus representing the energetic dependency of the syntrophic interactions reported in this study. The enhanced dechlorination observed in this study between *D. mccartyi* strain CBDB1 and partners supports previous observations on the impact of syntrophic partners in organochlorine respiring cultures (He et al., 2007; Men et al., 2012; Yan et al., 2012). The fact that strain CBDB1 shows higher activity in syntrophic partnership with *Desulfovibrio, Syntrophobacter*, and *Geobacter* lineages in the presence of exogenous VB_12_ represents a proof of concept for a bioreactor in which HCB could be reduced to less persistent oxidisable compounds (DCB, TCB) using lactate, propionate or acetate as energy sources which are cost effective and safe compared to H_2_.

**Table 4:** Hydrogen-releasing and hydrogen-consuming reactions occurring in co-cultures.

**HYDROGENOGENIC REACTIONS**	
**C_3_H_6_${\text{O}}_{3}^{-}$+ 2 H_2_O → CH_3_COO ^−+^${\text{HCO}}_{3}^{-}$+ H^+^ + 2H_2_**	**ΔG^0′^ = − 8.4 kJ/reaction**
**CH_3_CH_2_COO^−+^ 3 H_2_O → CH_3_COO^−+^${\text{HCO}}_{3}^{-}$+ 3 H_2_**	**ΔG^0′^ = + 76.1 kJ/reaction**
**CH_3_COO^−+^ 2 H_2_O → 2 ${\text{HCO}}_{3}^{-}$+ 4 H_2_ + H^+^**	**ΔG^0′^ = + 104.6 kJ/reaction**
**HYDROGEN-CONSUMING REACTIONS**	
**C_6_Cl_6_ + H_2_ → C_6_HCl_5_ + H^+^ + Cl^−^**	**ΔG^0′^ = − 171.4 kJ/reaction**
**C_6_Cl_6_ + 4 H_2_ → C_6_H_4_Cl_2_ + 4 H^+^ + 4 Cl^−^**	**ΔG^0′^ = − 447 kJ/reaction**

From previous work, in addition to supplying electron donor (H_2_), the robust growth of *D. mccartyi* strain 195 in syntrophic co-culture appears to stem from acetate supply by *D. vulgaris*, along with potential benefit from proton translocation, cobalamin-salvaging and amino acid biosynthesis as evidenced by gene expression analysis (Men et al., 2012). The impact of an incomplete Wood-Ljungdahl pathway i.e. the absence of CO dehydrogenase in the *D. mccartyi* genome, may also explain the more robust growth in co-culture and therefore further underpins the syntrophic interaction (Zhuang et al., 2014; Mao et al., 2015). *D. mccartyi* strain 195 was inhibited in pure culture by CO accumulating from acetyl-CoA cleavage due to the lack of CO dehydrogenase in *D. mccartyi* strain 195 (Zhuang et al., 2014).

In this study the inhibitory effect of CO on both dechlorination activity and cell growth of *D. mccartyi* strain CBDB1 was described. The adverse effect of CO toxicity on strain CBDB1 was mitigated by the presence of any of the tested syntrophic partners, (i.e. *Desulfovibro vulgaris, Syntrophobacter fumaroxidans*, or *Geobacter lovleyi*), which are capable of consuming CO (Davidova et al., 1994; Ragsdale, 2004; Diender et al., 2015). Other work using ^13^C-labeling and bioinformatic analysis confirmed that Bacteria and Archaea exist in consortia as CO-oxidizing organisms capable of gaining additional energy via coexistence with *D. mccartyi* while simultaneously enhancing growth of *D. mccartyi* (Zhuang et al., 2014). The results from the present study support and broaden previous observations of metabolic exchange between *D. mccartyi* and syntrophic partners in dechlorinating microbial environments (Zhuang et al., 2014; Mao et al., 2015). In a pure culture bioreactor based on CBDB1 activity an alternative approach to removal of CO would need to be devised.

The theoretical maximum interspecies distances for strain CBDB1 and partners were calculated based on the observed substrate oxidation rate. These estimates showed that proximity is more essential for syntrophic acetate and propionate oxidation compared to lactate oxidation. In the present study, cell aggregation was not observed in co-culture. When strain CBDB1 grew with *D. vulgaris* in co-culture, the maximum interspecies distance (178 μm) allowed H_2_ transfer between these bacteria without multi-lineage aggregate (flocculate) formation, whilst the other two co-cultures were close to the theoretical limit for H_2_ transfer over distance suggesting a reduction in distance between cells would lead to higher dichlorination activity. This result is consistent with observations by Mao et al. (2015) that aggregation was not established in co-cultures of strain 195 and *Desulfovibrio vulgaris* while it did occur in *Syntrophomonas wolfei* and strain 195 co-culture. Previous studies reported that syntrophic co-cultures form cell aggregates during the growth for optimal H_2_ transfer (Schink and Thauer, 1988; Stams et al., 2012; Felchner-Zwirello et al., 2013; Mao et al., 2015) or created biofilms in membrane bioreactors (Chung and Rittmann, 2008; Ziv-El et al., 2012). The cell aggregates reduce interspecies H_2_ transfer distance. In a bioreactor context, these data suggest approaches to stimulate flocculation or suspended biofilm formation would be beneficial.

In co-culture with CBDB1, *G. lovleyi* was hypothesized to provide cobalamin to support dechlorination of HCB as in previous studies on co-cultures of *G. lovleyi* and *D. mccartyi* strain 195 (Yan et al., 2012). However, there was only 0.006 ± 0.01 μg/L of extracellular cobalamin detected in CBDB1/GBL co-culture, while in the other co-cultures cobalamins were under the limit detection of the assay used. The amount of cobalamin detected was much lower than the requirement of cobalamin (50 μg/L) for maximal reductive dechlorination rates in pure CBDB1 culture (Adrian et al., 2000). Hence, syntrophic growth of *D. mccartyi* strain CBDB1 still requires exogenous cyanocobalamin supply for maximum HCB respiration rates. This suggests cyanocobalamin would be required as an amendment for a bioreactor application with significant implications with respect to cost.

Proteomics plays a key role in exploring adaptive responses of microbes to environments or interactions among microorganisms (Wang et al., 2016). Previous proteomic studies have evaluated the effects of long-term syntrophic growth in oxidation of butyrate (Schmidt et al., 2013; Sieber et al., 2015) or methanogenic consortia in degradation of terephthalate (Wu et al., 2013). Additionally, Men et al. (2012) explored protein expression during syntrophic interactions between *D. mccartyi* strain 195 and *Desulfovibrio vulgaris* strain Hildenborough dechlorinating trichloroethene.

In the present study, almost all ribosomal proteins in strain CBDB1 were found to be more abundant in the co-culture than in the isolate. Ribosomal proteins are related to cell growth indicating a higher rate of protein synthesis, resulting in a faster growth rate in the co-culture compared with the pure culture. This was congruent with our growth data. Similar to the observations of Men et al. (2012), the higher abundance of three CBDB1 hydrogenase proteins (VhuA, HymB, and HymC) and lower abundance of proteins related to electron transport such as NADH, oxidoreductase complexes as well as HymS in co-culture compared to in isolation suggested that syntrophic growth involves different H_2_ transfer systems than those used when H_2_ is supplied exogenously.

Another CBDB1 protein found with high abundance in co-culture was formate dehydrogenase, encoded by the *cbdbA195* gene. This protein is expected to have another function other than formate utilization found in both *D. mccartyi* strains CBDB1 and 195 (Adrian et al., 2007; Morris et al., 2007). Recently, Kublik et al. (2016) revealed that this formate dehydrogenase-like enzyme highly expressed in *D. mccartyi* is a putative molybdopterin containing oxidoreductase that co-localizes with the active subunit of the reductive dehalogenase (RdhA). This may explain the high abundance of these two proteins detected in co-cultures where favorable growth conditions lead to more respiration. These results support the only previous proteomic analysis of ORB syntrophic growth with *D. mccartyi* strain 195 and *D. vulgaris* with TCE dechlorination (Men et al., 2012).

When comparing proteins detected in *G. lovleyi* in isolation and GBL/CBDB1 co-culture, there were two proteins detected exclusively in the latter: peptidylprolylisomerase and histidinol dehydrogenase. Histidinol dehydrogenase is responsible for catalyzing the reaction: L-histidinol + NAD^+^ = L-histidine + NADH + H^+^, while peptidylprolylisomerase plays the role of PceT, identified to be a trigger factor-like protein that functions as a dedicated chaperone for PceA (Maillard et al., 2011). The reason why these two proteins were only detected in co-culture and not expressed in *G. lovleyi* pure culture is unclear.

In the present study, no cobalamin biosynthesis proteins from *G. lovleyi* were detected and no differences in expression of cobalamin transport proteins were detected when comparing the co-culture with *G. lovleyi* in pure culture. These results might explain why cobalamin production by *G. lovleyi* was unable to support the GBL/CBDB1 co-culture. The observation that proteins related to acetate oxidation through the citric acid cycle (malate dehydrogenase, isocitratedehyrogenase and citrate synthase) were more abundant in co-culture than in the isolate is consistent with the stimulation of acetate oxidation by syntrophic growth. The lack of flagellin and lower expression of chemotaxis sensory transducer in co-culture supports the idea that syntrophic growth of CBDB1 and *G. lovleyi* occurs via interspecies H_2_ transfer.

Cross-inhibition of co-contaminants and self-inhibition by biodegradation products in the environment has been widely reported (Chan et al., 2011; Schiffmacher et al., 2016). DCB and TCB amended in pure CBDB1 cultures were found to be inhibitory. The presence of GAC in CBDB1 co-culture headspace reduced the inhibitory effect of HCB daughter products on cell growth and dechlorination activity. Application of GAC to remove chlorinated compounds has been studied widely from water to soil in contaminated environments (Pavoni et al., 2006; Choi et al., 2009; Kjellerup et al., 2014; Dang et al., 2016). The data presented here suggests this approach could serve as an effective strategy in bioreactor applications with GAC mounted in a reactor headspace serving to continuously remove DCB and TCB to prevent daughter product inhibition.

## Conclusions

Despite the ubiquity of syntrophic processes in anoxic environments, little is known about the mechanisms by which syntrophic consortia regulate their metabolism, such as the cross-supply of cobalamins or proteomic insights into syntrophic growth during organohalide respiration. Here we provide the first description of syntrophic growth of *D. mccartyi* strain CBDB1 and partners (*Desulfovibrio vulgaris, Syntrophobacter fumaroxidans* and *Geobacter lovleyi*) for exogenous provision of H_2_, acetate and cobalamin for the reductive dechlorination of HCB. HCB dechlorination rates were enhanced two- to three-fold compared to pure cultures of *D. mccartyi* strain CBDB1. In co-cultures, acetate and H_2_ were supplied from substrate oxidation by syntrophic partners, whilst amendment of exogenous cobalamin was necessary in all cultures to achieve maximal rates. Superior growth and dechlorination by strain CBDB1 in co-culture may be explained by the consumption of CO, preventing the adverse effect of CO toxicity by the presence of any of its syntrophic partners. Additionally, proteomic analysis in co-culture revealed an abundance of ribosomal proteins, reductive dehalogenase, hydrogenase proteins, and formate dehydrogenase, and down-regulation of proteins related to electron transport. The GAC application and results obtained from this study were the first investigation on GAC application in attempt to mitigate the inhibitory effect of HCB daughter products and thus enhance HCB dechlorination activity. This approach is relevant to bioreactor applications for HCB remediation with a potential low cost and easy application.

## Author contributions

AC conceived, designed, analysed and executed the experimental work as well as drafted the manuscript. ML, LA, and MM conceived, designed, performed the data analysis, and the interpretation. ML, LA, and MM drafted the manuscript.

### Conflict of interest statement

The authors declare that the research was conducted in the absence of any commercial or financial relationships that could be construed as a potential conflict of interest.
