# Supplementary material for: Syntrophic Partners Enhance Growth and Respiratory Dehalogenation of Hexachlorobenzene by Dehalococcoides mccartyi Strain CBDB1
Source: Front Microbiol. 2018 Aug 22;9:1927. doi: 10.3389/fmicb.2018.01927 (PMC6113397; doi:10.3389/fmicb.2018.01927)
Supplement: Supplementary file 1 [file Table_1.DOCX]

**Table S1.** Parameters in Fick's equation and allowed interspecies distance calculation ^(a)^

|  |  |  |  |
| --- | --- | --- | --- |
|  | *D. vulgaris* with strain CBDB1 | *G. lovleyi* with strain CBDB1 | *S. fumaroxidans* with strain CBDB1 |
|  | on lactate | on acetate | on propionate |
| A_syn_ (μm^2^) ^(b)^ | 1.3 | 2.0 | 2.1 |
| Δcell _syn_ (day60-day0)^(c)^ | 6.5 x 10^7^ | 1.9 x 10^8^ | 2.7 x 10^7^ |
| C_H2-syn_ μM | 8.7 ± 0.2 | 1.6 ± 0.08 | 5.6 ± 0.1 |
| J_H2_ (pmol m^-2^ s^-1^) | 19.9 x 10^5^ | 4.97 x 10^5^ | 1.9 x 10^5^ |
| d_syn-strain CBDB1_(μm) | 178 | 10.1 | 18.6 |
|  |  |  |  |

^a^The values were calculated in a time interval from day 0 to day 60, at 307.15 K

^b^Surface area of *G. lovleyi, D. vulgaris* and *S. fumaroxidans* based on cells diameters of 0.4 , 0.25 and 0.25 μm respectively and lengths of 1.4, 1.5 and 2.5 μm respectively.

^c^Syntroph cell number increase from day 0 to day 60.

Calculation of allowed interspecies distance in syntrophic partners and *Dehalococcoides mccartyi* strain CBDB1 co-culture by using Fick's diffusion law. Data from this table was calculated following Mao et al. (2015).

Assuming the cells were evenly dispersed in the bottle, the average cell-cell distance in GLB/CBDB1 co-culture can be calculated as below:

1 cm

10^4^ µm

1 cm

3 1,34 x 10^8^

D_cell-cell_ = x = 19.5 µm

Total cell number of *D. mccartyi* strain CBDB1 and *G. lovleyi* in co-culture was 1.34 x 10^8^ cell/ml at day 60 of incubation. Hence, the estimation of cell-cell distance in dispersed cell is 19.5 µm. This distance is larger than the predicted distance (10.1 µm) that can support interspecies H_2_ transfer at the measured acetate oxidation rate. Therefore, in order to accomplish syntrophic acetate oxidation at the rate observed, the average interspecies distance should be less than the distance between randomly dispersed cells.
