# Supplementary material for: Syntrophic Partners Enhance Growth and Respiratory Dehalogenation of Hexachlorobenzene by Dehalococcoides mccartyi Strain CBDB1
Source: Front Microbiol. 2018 Aug 22;9:1927. doi: 10.3389/fmicb.2018.01927 (PMC6113397; doi:10.3389/fmicb.2018.01927)
Supplement: Supplementary file 2 [file Table_2.DOCX]

**Table S2.** *D. mccartyi* and *G. lovleyi* proteins upregulated in co-culture.

| **NCBI locus** | **Predicted functions** | ***D. mccartyi*** | ***G. lovleyi*** |
| --- | --- | --- | --- |
| cbdbA84 | putative reductive dehalogenase | 33 | _ |
| cbdbA80 | putative reductive dehalogenase | 14 | _ |
| cbdbA195 | formate dehydrogenase, major subunit | 8.2 | _ |
| cbdbA960 | translation elongation factor Tu | 5.6 | _ |
| cbdbA1358 | chaperone protein DnaK | 2 | _ |
| cbdbA1393 | co-chaperonin GroEL | 2 | _ |
| cbdbA727 | hypothetical protein | 2 | _ |
| cbdbA597 | hydrogenase, group 3, VhuA subunit | 2 | _ |
| cbdbA684 | putative [Fe] hydrogenase, HymB subunit | 2 | _ |
| cbdbA685 | putative [Fe] hydrogenase, HymC subunit | 2 | _ |
| Glov_0477 | conserved hypothetical protein | _ | 8.6 |
| Glov_1625 | malate dehydrogenase | _ | 6.6 |
| Glov_0475 | alkyl hydroperoxide reductase | _ | 5.5 |
| Glov_2929 | 60 kDa chaperonin | _ | 4.9 |
| Glov_1926 | histone family protein DNA-binding protein | _ | 4.8 |
| Glov_1629 | pyruvate ferredoxin/flavodoxin oxidoreductase | _ | 4.4 |
| Glov_1624 | isocitrate dehydrogenase, NADP-dependent | _ | 4.4 |
| Glov_1339 | DNA-directed RNA polymerase subunit beta | _ | 4.4 |
| Glov_1379 | citrate (Si)-synthase | _ | 4.4 |
| Glov_0345 | acetyl-CoA hydrolase | _ | 4.4 |
| Glov_3137 | NADH-quinone oxidoreductase subunit B | _ | 3.9 |
| Glov_1119 | ATP phosphoribosyltransferase | _ | 3.9 |
| Glov_1340 | DNA-directed RNA polymerase subunit beta' | _ | 3.3 |
| Glov_1216 | 6,7-dimethyl-8-ribityllumazine synthase | _ | 3.1 |
| Glov_2213 | succinate dehydrogenase  fumarate reductase, flavoprotein subunit | _ | 2.9 |
| Glov_3058 | lipoprotein, putative | _ | 2.9 |
| Glov_2695 | adenylosuccinate lyase | _ | 2.9 |
| Glov_2639 | histone family protein DNA-binding protein | _ | 2.9 |
| Glov_1628 | thiamine pyrophosphate domain protein TPP-binding | _ | 2.8 |
| Glov_1214 | homoserine dehydrogenase | _ | 2.6 |
| Glov_2216 | FAD-dependent pyridine nt-disulphide oxidoreductase | _ | 2.6 |
| Glov_1927 | ATP-dependent Clp protease ATP-binding subunit ClpX | _ | 2.6 |
| Glov_0751 | Anti-sigma factor antagonist | _ | 2.6 |
| Glov_3124 | MglA protein | _ | 2.6 |
| Glov_2490 | protein translocase subunit SecA | _ | 2.4 |
| Glov_2709 | elongation factor Ts | _ | 2.4 |
| Glov_3261 | biotin/lipoyl attachment domain-containing protein | _ | 2 |
| Glov_1606 | nitrogen fixation protein NifU | _ | 2 |
| Glov_1607 | cysteine desulfurase NifS | _ | 2 |
| Glov_1727 | CTP synthase | _ | 2 |
| Glov_1343 | elongation factor G | _ | 2 |
| Glov_3161 | protein RecA | _ | 2 |
| Glov_3154 | alanine--tRNA ligase | _ | 2 |
| Glov_3325 | 2-isopropylmalate synthase | _ | 2 |
| Glov_3262 | propionyl-CoA carboxylase | _ | 2 |
| Glov_1617 | phosphoglycerate kinase | _ | 2 |
| Glov_0665 | Cell division protein | _ | 2 |
| Glov_1338 | 50S ribosomal protein L7/L12 | _ | 2 |
| Glov_2145 | ribosomal protein S1 | _ | 2 |
| Glov_3260 | methylmalonyl-CoA mutase, large subunit | _ | 2 |
