# Supplementary figures and images for: Syntrophic Partners Enhance Growth and Respiratory Dehalogenation of Hexachlorobenzene by Dehalococcoides mccartyi Strain CBDB1
Source: Front Microbiol. 2018 Aug 22;9:1927. doi: 10.3389/fmicb.2018.01927 (PMC6113397; doi:10.3389/fmicb.2018.01927)

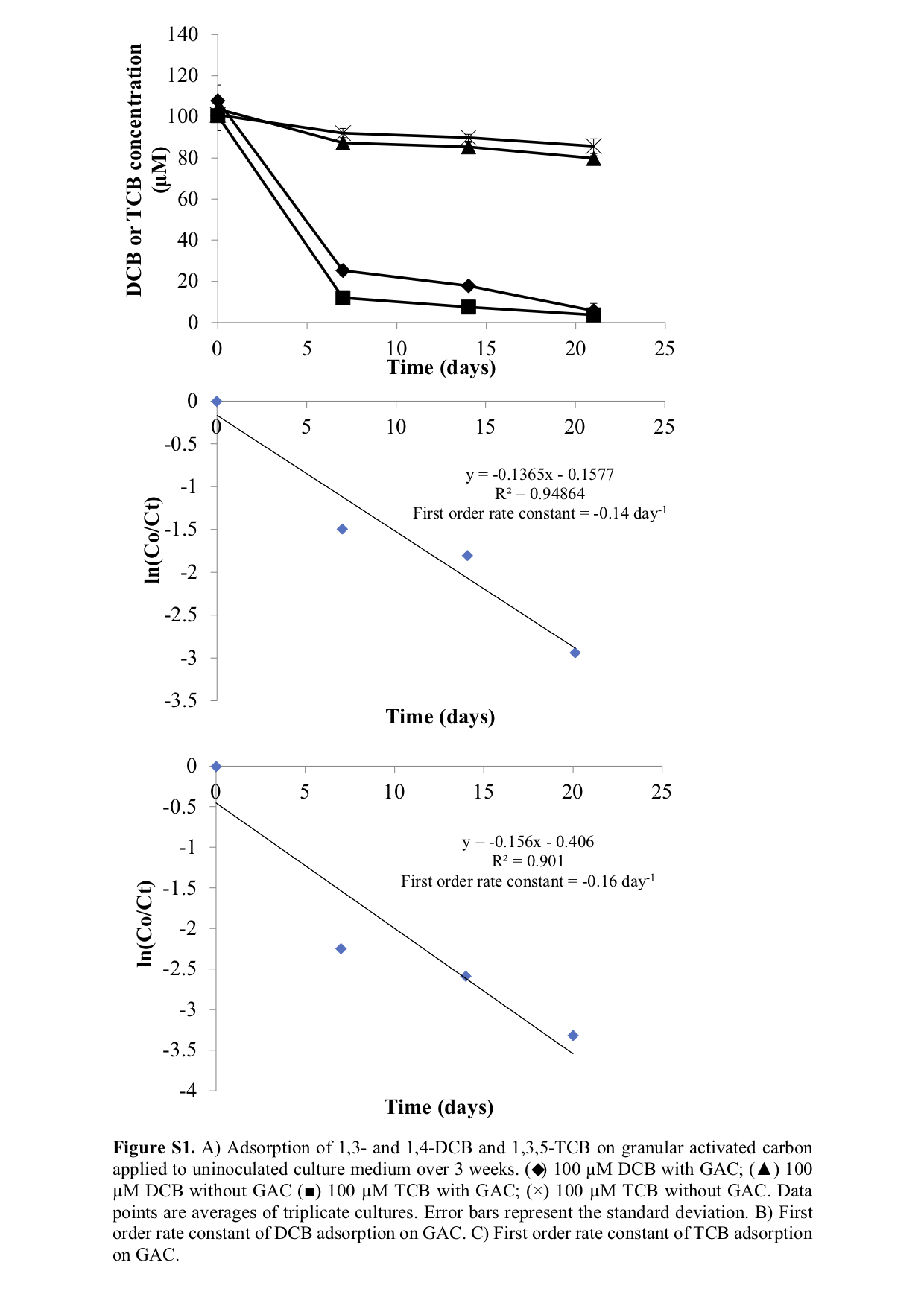

Supplement: Supplementary file 4 [file Image_1.TIFF]

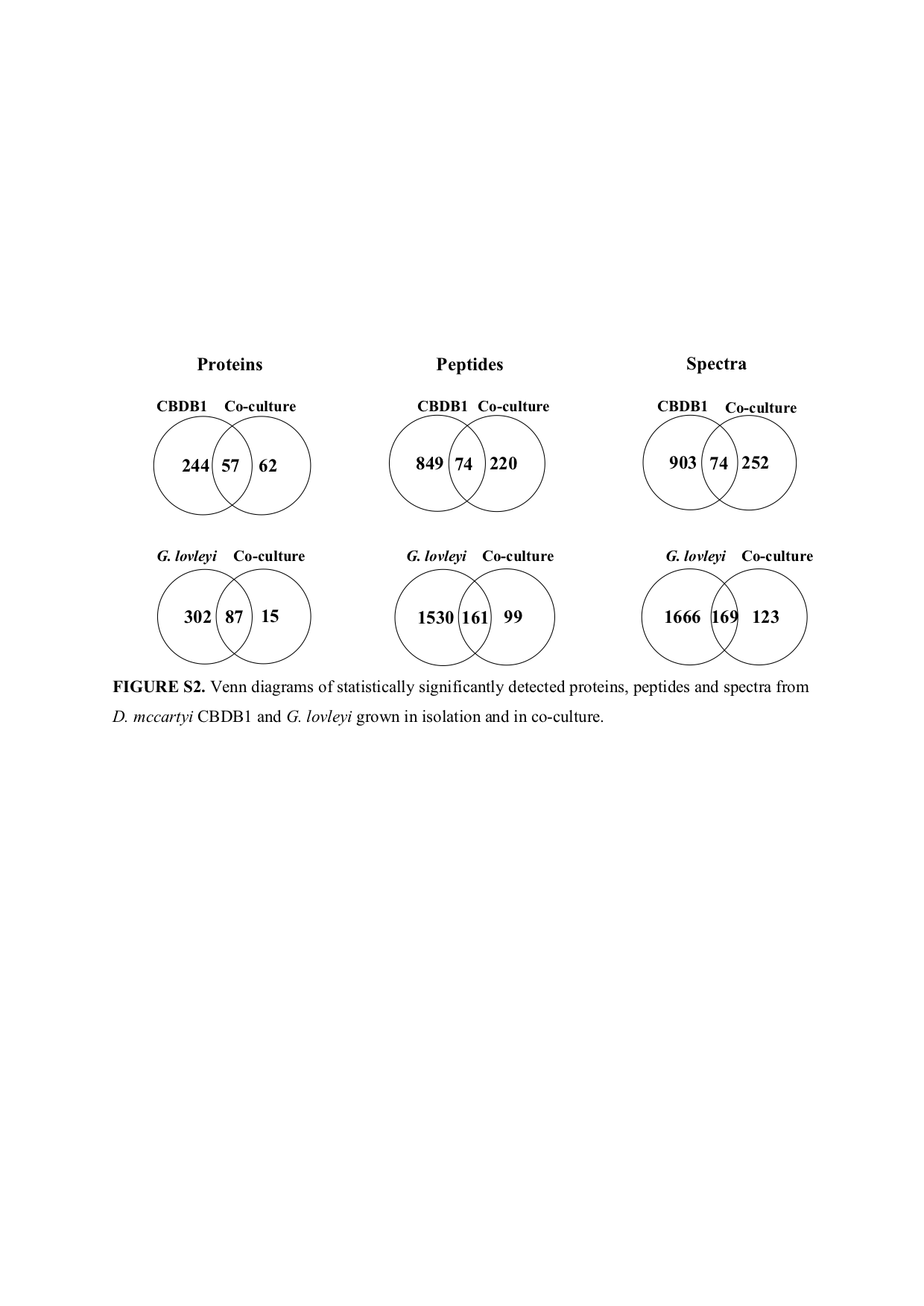

Supplement: Supplementary file 5 [file Image_2.TIFF]

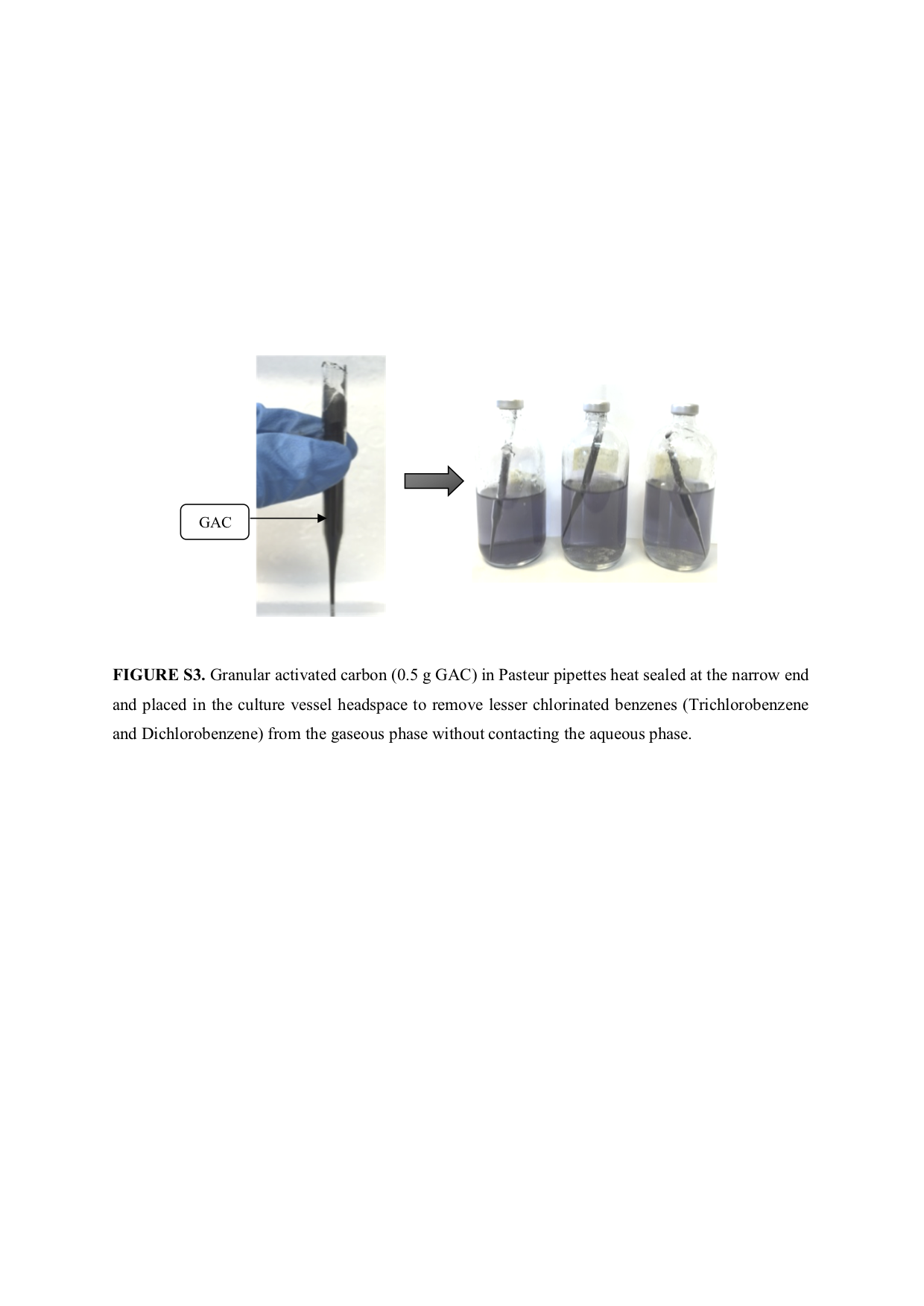

Supplement: Supplementary file 6 [file Image_3.TIFF]
